# Association Between Glucagon-Like Peptide-1 (GLP-1) Receptor Agonist Use and Blood Pressure Levels in Nondiabetic Obese Adults: A Cross-Sectional Analysis

**DOI:** 10.7759/cureus.108646

**Published:** 2026-05-11

**Authors:** Moses C Odoeke, Steve Okwu, Ekapong Bassey, Akinyele Oladimeji, Thy-Will D Aboflah, Stella Adelola, Michael O Ewejobi, Nnaemeka H Ugwuchukwu, Daad G Alrowaili, Okelue E Okobi

**Affiliations:** 1 Internal Medicine, University of Toledo, Toledo, USA; 2 Cardiothoracic Surgery, Beacon Hospital, Dublin, IRL; 3 General Medicine, Igbinedion University Okada, Okada, NGA; 4 Family Medicine, Alberta Health Services, Edmonton, CAN; 5 Internal Medicine, The Bank Hospital, Accra, GHA; 6 Medicine, International University of the Health Sciences, Basseterre, KNA; 7 Oncology, Antrim Area Hospital, Antrim, GBR; 8 Genetics and Human Genetics, Howard University, Washington, D.C., USA; 9 Family Medicine, Larkin Community Hospital Palm Springs Campus, Hialeah, USA; 10 Family Medicine, IMG Research Academy and Consulting LLC, Homestead, USA

**Keywords:** blood pressure, glp-1 receptor agonist, hypertension, nhanes, nondiabetic adults, obesity

## Abstract

Background and aim

Obesity is strongly linked to elevated blood pressure and increased cardiovascular risk. Glucagon-like peptide-1 (GLP-1) receptor agonists have gained attention for their role in weight management and potential cardiovascular benefits. Evidence in nondiabetic populations remains limited. The aim of this study was to descriptively evaluate the association between GLP-1 receptor agonist use and systolic and diastolic blood pressure levels among nondiabetic adults with obesity.

Methods

This cross-sectional study used data from the National Health and Nutrition Examination Survey (NHANES) 2017 to March 2020 prepandemic cycle, selected to ensure use of the most recent complete data prior to disruptions related to the COVID-19 pandemic. Adults aged 18 years or older with obesity were included, and individuals with self-reported diabetes were excluded. GLP-1 receptor agonist use was identified from prescription medication data, with a very small number of exposed participants in the analytic sample. Blood pressure was assessed using standardized examination measures. A complete-case approach was applied, whereby participants with missing data on any variables included in the analysis were excluded to ensure a consistent analytic sample. Analyses incorporated NHANES examination sample weights to produce nationally representative estimates and accounted for the complex survey design by including stratification and primary sampling units to ensure correct variance estimation. Descriptive statistics were used to compare characteristics by exposure.

Results

Lower systolic blood pressure was observed among GLP-1 users compared with nonusers (107.2 vs 122.1 mmHg), and diastolic blood pressure was also lower (74.4 vs 76.9 mmHg). The proportion of hypertension was lower among GLP-1 users, 5,814 (14.1%), compared with nonusers, 25,207,405 (36.5%). The extremely small number of exposed participants (n = 2) limited further statistical analysis and inference.

Conclusions

Lower blood pressure levels were observed among GLP-1 receptor agonist users compared with nonusers in this population. These findings are descriptive and reflect observed differences in blood pressure within the analytic sample, without implying a causal relationship. Further studies using datasets with greater representation of GLP-1 receptor agonist use in nondiabetic populations are needed to better characterize this relationship.

## Introduction

Obesity is a significant and increasing global public health issue and is closely linked to an augmented risk of cardiovascular disease, high blood pressure, and premature death [1]. High blood pressure is one of the highest-risk factors that can be modified among obese individuals and is associated with adverse cardiovascular outcomes [2,3]. Traditional strategies for controlling blood pressure (e.g., lifestyle modification and antihypertensive medications) continue to be of paramount importance; however, there is increasing interest in the development of alternative therapeutic options that can simultaneously address both weight loss and cardiovascular/metabolic risk [4,5].

The pharmacologic class of glucagon-like peptide-1 (GLP-1) receptor agonists has been shown to be very effective for weight loss in the obese population, especially among those with obesity associated with type 2 diabetes mellitus [6]. GLP-1 receptor agonists have unique mechanisms of action that produce weight loss, including appetite suppression, delayed gastric emptying, and changes in the sensation of fullness [7,8]. More recently, there is also evidence that GLP-1 receptor agonists can provide cardiovascular benefits, such as reduction of blood pressure, through weight loss, enhanced endothelial function, diuresis (additional loss of water), and alterations in neurohormones [9].

Although there are many studies demonstrating that GLP-1 receptor agonists reduce blood pressure in people with diabetes, much less is known about the effects of GLP-1 receptor agonists on blood pressure in the nondiabetic obese population [10,11]. This group of patients is particularly important for early intervention, as obesity-related hypertension frequently precedes the development of clearly defined type 2 diabetes mellitus and significantly increases the long-term risk of developing cardiovascular disease [12]. Determining whether there is any benefit to using GLP-1 receptor agonists for lowering blood pressure in nondiabetic obese patients will help develop broader therapeutic approaches beyond managing glycemia in the nondiabetic obese population [13].

The regulation of blood pressure in individuals with obesity is influenced by a large number of contributing factors, each with a complex relationship to the others [14]. These include adiposity, insulin resistance, inflammation, and vascular dysfunction [15]. Therefore, evaluation of the real-world effects of GLP-1 receptor agonist use requires sufficient data regarding both clinical measurements and demographic and behavioral factors related to medication use [16].

There is currently growing interest in the cardiometabolic benefits of GLP-1 receptor agonists; however, there continues to be a lack of population-level evidence evaluating the association between blood pressure reduction and GLP-1 receptor agonist use specifically among obese individuals without diabetes [17]. Many existing studies have been conducted within controlled clinical environments, with little to no focus on people in real-world settings who may differ in their use of GLP-1 receptor agonists and/or individual characteristics from those who participate in controlled clinical trials [18].

This study employed the National Health and Nutrition Examination Survey (NHANES) to provide more comprehensive results and findings. The NHANES dataset offers a unique opportunity to conduct analyses based on standardized clinical measurements (blood pressure and anthropometric measurements) and quantitative data collected from questionnaires regarding medication use, lifestyle behaviors, and comorbid conditions [19].

The main objective of this study was to descriptively evaluate the association between GLP-1 receptor agonist use and blood pressure levels among nondiabetic obese adults using NHANES 2017-2020 data [20]. The findings of this study will add to the existing literature regarding how GLP-1 receptor agonist use may reduce cardiovascular risk and assist in making clinical decisions related to the treatment of obesity.

## Materials and methods

Study design and data source

This study employed a cross-sectional design using publicly available data from the NHANES 2017 to March 2020 prepandemic cycle, selected to ensure use of the most recent complete data prior to disruptions related to the COVID-19 pandemic [20]. NHANES is a nationally representative survey conducted by the National Center for Health Statistics using a complex, multistage probability sampling design to assess the health and nutritional status of the civilian, noninstitutionalized United States population. Data are collected through household interviews and standardized physical examinations conducted in mobile examination centers. This study utilized demographic, examination, laboratory, and prescription medication files. Blood pressure measurements and anthropometric data were obtained from the examination component, while medication use was derived from the prescription medication files.

Study population

The initial merged dataset included 15,560 participants with available demographic information, among whom 68 individuals were identified as using GLP-1 receptor agonists based on prescription medication data. Participants younger than 18 years were excluded, followed by restriction to individuals with obesity defined as a BMI of at least 30 kg/m². Nondiabetic status was defined based on self-reported physician diagnosis using the diabetes questionnaire variable DIQ010, and individuals reporting a diagnosis of diabetes were excluded [20]. These eligibility criteria substantially reduced the number of participants using GLP-1 receptor agonists, reflecting prescribing patterns during the study period. After applying these restrictions, 3,705 participants remained, including two individuals reporting GLP-1 receptor agonist use, reflecting the low prevalence of GLP-1 receptor agonist use among nondiabetic obese adults during the study period and supporting the descriptive nature of the analysis.

A complete-case approach was then applied, excluding participants with missing data on key variables, including age, sex, BMI, blood pressure measurements, poverty-income ratio, education level, hypertension status, and GLP-1 receptor agonist use. This resulted in a final analytic sample of 2,269 participants, representing a weighted population of 69,176,384 individuals.

Variables and measures

The primary exposure was GLP-1 receptor agonist use, derived from the prescription medication files. Medication names were converted to lowercase and searched to identify GLP-1 receptor agonists, including liraglutide, semaglutide, dulaglutide, exenatide, lixisenatide, and albiglutide. A binary variable was created indicating use if any of these medications were reported. The outcomes of interest were systolic and diastolic blood pressure measured in the examination component. Up to four measurements were obtained per participant, and mean systolic and diastolic blood pressure values were calculated using available readings. Covariates included age in years, sex, race or ethnicity, BMI, poverty-income ratio, education level, and hypertension status based on questionnaire data. Race or ethnicity was categorized using standard NHANES groupings. Education was categorized as less than high school, high school graduate, or college and above. Antihypertensive medication use and glycated hemoglobin were initially considered but were excluded from the final analysis because of substantial missingness.

Missing data

Missingness was assessed for all variables prior to analysis to evaluate data completeness and guide analytic decisions. Several variables had moderate levels of missing data, including poverty-income ratio (15.98%), BMI (20.67%), and systolic and diastolic blood pressure (each 26.29%), while education had lower missingness (4.40%). Antihypertensive medication use had substantial missingness (66.10%) and was excluded from the analysis to avoid introducing bias from incomplete data.

A complete-case approach was applied by excluding participants with missing data on variables included in the final analysis. This approach ensured a consistent analytic sample across all descriptive comparisons, which is particularly important given the already limited number of exposed participants. While this method may reduce sample size and introduce potential selection bias if missingness is not random, it was chosen to maintain internal consistency and interpretability of results within this descriptive analysis. Variables with minimal missingness included age, sex, race or ethnicity, survey design variables, GLP-1 receptor agonist use, and diabetes status.

Statistical analysis

All analyses accounted for the complex survey design of NHANES using examination sample weights, strata, and primary sampling units. The combined prepandemic examination weights were applied by appropriately adjusting NHANES cycle-specific weights to account for the 2017-March 2020 combined dataset, ensuring that estimates were nationally representative of the U.S. population. Descriptive statistics were used to summarize participant characteristics by GLP-1 receptor agonist use. Continuous variables were reported as weighted means with SDs, and categorical variables were reported as weighted counts and column percentages. Due to the extremely small number of participants using GLP-1 receptor agonists in the analytic sample, inferential analyses were not emphasized, and no hypothesis testing was performed. Graphical presentation included the distribution of age in the analytic sample to provide additional descriptive context. All analyses were conducted using Stata version 18 (StataCorp LLC, College Station, TX, USA).

Ethical considerations

NHANES protocols are approved by the National Center for Health Statistics Research Ethics Review Board, and all participants provide written informed consent prior to participation. The present study used publicly available, deidentified data and did not require additional institutional review board approval.

## Results

To address the study objective of evaluating the association between GLP-1 receptor agonist use and blood pressure levels, Table 1 presents weighted comparisons of systolic and diastolic blood pressure between users and nonusers, along with other participant characteristics.

**Table 1:** Weighted characteristics of the study population by GLP-1 receptor agonist use (weighted n = 69,176,384; unweighted n = 2,269) Values are presented as weighted means with SDs for continuous variables and weighted counts with column percentages for categorical variables. Estimates account for the complex survey design of NHANES using examination sample weights, strata, and primary sampling units. GLP-1 receptor agonists include liraglutide, semaglutide, dulaglutide, exenatide, lixisenatide, and albiglutide. p-values and statistical tests were not reported because of the extremely small number of GLP-1 users (n = 2), which limits the validity of survey-adjusted comparisons. GLP-1, glucagon-like peptide-1; NHANES, National Health and Nutrition Examination Survey

Variable	GLP-1 agonist nonusers (N = 69,135,179)	GLP-1 agonist users (N = 41,205)
Age (years), mean ± SD	46.4 ± 16.3	42.1 ± 0.4
BMI (kg/m²), mean ± SD	36.1 ± 5.9	35.8 ± 1.6
Systolic blood pressure (mmHg), mean ± SD	122.1 ± 16.4	107.2 ± 22.6
Diastolic blood pressure (mmHg), mean ± SD	76.9 ± 10.6	74.4 ± 13.3
Poverty-income ratio, mean ± SD	3.10 ± 1.62	2.97 ± 1.00
Sex, n (%)		
Male	32,931,836 (48.0%)	5,814 (14.1%)
Female	36,203,342 (52.0%)	35,391 (85.9%)
Race/ethnicity, n (%)		
Mexican American	6,337,468 (9.2%)	0 (0.0%)
Other Hispanic	5,006,263 (7.2%)	0 (0.0%)
Non-Hispanic White	44,399,840 (64.2%)	35,391 (85.9%)
Non-Hispanic Black	8,798,952 (12.7%)	5,814 (14.1%)
Non-Hispanic Asian	1,328,275 (1.9%)	0 (0.0%)
Other race	3,264,382 (4.7%)	0 (0.0%)
Education, n (%)		
Less than high school	6,270,238 (9.1%)	0 (0.0%)
High school graduate	19,874,957 (28.7%)	0 (0.0%)
College or above	42,989,983 (62.2%)	41,205 (100.0%)
Hypertension, n (%)		
No	43,927,774 (63.5%)	35,391 (85.9%)
Yes	25,207,405 (36.5%)	5,814 (14.1%)

The results from the table above indicate that the weighted mean age was 46.4 ± 16.3 years among nonusers and 42.1 ± 0.4 years among GLP-1 users. The mean BMI was similar between groups, 36.1 ± 5.9 kg/m² in nonusers and 35.8 ± 1.6 kg/m² in users. Systolic blood pressure was lower among GLP-1 users, with a mean of 107.2 ± 22.6 mmHg compared with 122.1 ± 16.4 mmHg in nonusers, and diastolic blood pressure was also lower in users, 74.4 ± 13.3 mmHg compared with 76.9 ± 10.6 mmHg. Poverty-income ratio values were comparable between groups. The distribution of sex differed, with females accounting for 36,203,342 (52%) among nonusers and 35,391 (85.9%) among GLP-1 users. Non-Hispanic White participants represented the largest racial group in both categories. All GLP-1 users were classified as having a college-level education or higher. The proportion without hypertension was higher among GLP-1 users, 35,391 (85.9%), compared with 43,927,774 (63.5%) among nonusers.

These findings describe differences between groups; formal statistical comparisons were not conducted due to the extremely small number of GLP-1 receptor agonist users (n = 2), which limits the validity of survey-adjusted tests.

## Discussion

The present study examined the association between GLP-1 receptor agonist use and blood pressure levels among nondiabetic obese adults using nationally representative data. The descriptive findings showed lower mean systolic and diastolic blood pressure among GLP-1 users compared with nonusers. Systolic blood pressure averaged 107.2 mmHg among users and 122.1 mmHg among nonusers, while diastolic blood pressure was 74.4 mmHg and 76.9 mmHg, respectively. The distribution of hypertension was also lower among GLP-1 users. These observations are consistent with prior reports indicating that GLP-1 receptor agonists may be associated with modest reductions in blood pressure in populations with obesity [10,13]. Previous analyses of NHANES data have also demonstrated that obesity is strongly linked to adverse cardiovascular risk profiles, including elevated blood pressure, which provides context for the patterns observed in this study [1,2]. The current findings extend this evidence to a specific subgroup of nondiabetic obese adults, although the results remain descriptive in nature due to the limited number of exposed participants.

Current United States hypertension guidelines emphasize the importance of blood pressure control in individuals with obesity, with a target threshold of less than 130 over 80 mmHg for most adults at increased cardiovascular risk [4]. These guidelines highlight weight management, lifestyle modification, and pharmacologic therapy as key components of care. The lower blood pressure levels observed among GLP-1 users in this study align with the clinical goal of achieving improved cardiovascular profiles in individuals with obesity. National data have shown that blood pressure control remains suboptimal among adults with higher BMIs, further supporting the need for effective interventions in this population [3,19]. The descriptive differences observed in this study are in line with these recommendations, although the findings should not be interpreted as evidence of treatment effect.

Several biological mechanisms may explain the observed differences in blood pressure levels among GLP-1 users. GLP-1 receptor agonists are known to influence appetite regulation and energy balance through central and peripheral pathways, leading to weight reduction, which is closely linked to improvements in blood pressure [7,8]. In addition, these agents may exert direct cardiovascular effects, including improved endothelial function and modulation of vascular tone, which can contribute to lower blood pressure [9]. Obesity-related hypertension is driven by complex interactions involving sympathetic nervous system activation, renal sodium retention, and hormonal pathways [14,15]. Interventions that target weight and metabolic regulation may therefore be associated with improvements in blood pressure profiles. These mechanisms provide a plausible explanation for the descriptive patterns observed in this study without implying a causal relationship.

Strengths and limitations of the study

This study has several strengths and limitations. The use of NHANES data allows for nationally representative estimates and appropriate incorporation of survey weights, strata, and clustering. Standardized measurement of blood pressure and anthropometric variables enhances data quality and comparability across participants.

However, important limitations should be considered. The cross-sectional design limits temporal interpretation and precludes causal inference. Exposure to GLP-1 receptor agonists was based on self-reported medication use, which may introduce misclassification. Missing data led to the use of a complete-case approach, which may reduce generalizability and introduce selection bias if missingness is not random.

A major limitation is the extremely small number of exposed participants (n = 2) after applying study restrictions. This severely limits statistical power, precludes inferential analysis, and restricts the findings to descriptive comparisons. As a result, the observed differences in blood pressure should be interpreted with caution and cannot be considered evidence of an independent association.

Residual confounding is also likely. Several important factors that influence both GLP-1 receptor agonist use and blood pressure were not included or could not be fully accounted for, including antihypertensive medication use, duration and severity of obesity, baseline cardiovascular risk, healthcare access, prescribing patterns, and socioeconomic factors. Behavioral determinants such as diet, physical activity, and adherence to treatment were not assessed and may also influence blood pressure outcomes. The exclusion of antihypertensive medication use and glycated hemoglobin due to substantial missingness further limits adjustment for key clinical factors.

In addition, confounding by indication is a concern, as individuals prescribed GLP-1 receptor agonists may differ systematically from nonusers in ways related to underlying health status or clinical management. Reverse causation cannot be excluded, as differences in blood pressure may influence treatment patterns rather than result from medication use.

Given these limitations, the findings should be considered hypothesis-generating and reflective of patterns within the analytic sample rather than definitive evidence of association. Future research should utilize larger and more recent datasets or longitudinal designs with sufficient representation of GLP-1 receptor agonist use to allow for robust adjustment of confounders and more reliable evaluation of associations with blood pressure outcomes.

## Conclusions

This study highlights descriptive differences in blood pressure levels between GLP-1 receptor agonist users and nonusers among nondiabetic obese adults. Lower blood pressure values were observed among exposed individuals, though these findings remain descriptive and should be interpreted with caution. The results reflect patterns within a nationally representative population and align with current clinical interest in therapies that address both obesity and cardiovascular risk. The limited number of exposed participants restricted formal statistical evaluation and emphasizes the need for appropriate data sources. Future research should use datasets with greater representation of GLP-1 receptor agonist use in nondiabetic populations and incorporate longitudinal designs to better assess associations with blood pressure and related clinical outcomes.
